# Classification, diagnosis, and management of conjunctival lymphoma

**DOI:** 10.1186/s40662-019-0146-1

**Published:** 2019-07-27

**Authors:** Rebecca E. Tanenbaum, Anat Galor, Sander R. Dubovy, Carol L. Karp

**Affiliations:** Department of Ophthalmology, Bascom Palmer Eye Institute, University of Miami Miller School of Medicine, 900 NW 17th St., Miami, Miami, FL 33136 USA

**Keywords:** Conjunctiva, Conjunctival tumor, Extranodal marginal zone lymphoma, Lymphoma, Optical coherence tomography, Salmon patch

## Abstract

Lymphoma is a malignant lymphoproliferative tumor that can involve the conjunctiva. Approximately 5–15% of all extranodal lymphomas are found in the ocular adnexal region, with approximately 25% of those involving the conjunctiva. Ninety-eight percent of conjunctival lymphomas arise from B-lymphocytes. The most common subtype of conjunctival lymphoma is extranodal marginal zone lymphoma (80%), followed by follicular lymphoma (8%), diffuse large B-cell lymphoma (3%) and mantle cell lymphoma (3%). Natural killer and T cells (NK/T) are rare causes of lymphoma. While most conjunctival lymphomas are localized to the ocular adnexa at the time of presentation, systemic examination and management are of key importance in the long-term care of the patient.

This review outlines the classification, etiology, presentation, diagnosis, and management of conjunctival lymphoma. The novel use of high resolution optical coherence tomography, both as a diagnostic tool and as a means for ongoing evaluation during treatment, is illustrated. Treatment options discussed include external beam radiation, chemotherapy, immunotherapy, antibiotic therapy, and combination regimens. Future investigation of the etiology and pathogenesis of conjunctival lymphoma is expected to reveal opportunities for innovative and individualized therapeutic agents. Collaboration between multiple disciplines is key in the advancement of the field.

## Background

The conjunctiva is an important site of extranodal lymphoma development, which comprises 25–30% of all lymphomatous disease [1, 2]. While less common than conjunctival squamous neoplasia or melanoma, conjunctival lymphoma accounts for a significant portion of ocular adnexal lymphomas (OALs) (25%) [3, 4]. If not detected or followed properly, the disease may progress systemically. Histologic subtype, as well as other characteristics such as laterality, is a critical predictor of prognosis and management. Many clinicians may not regularly encounter these tumors in practice, and thus feel uncomfortable managing the disease. This paper provides a comprehensive review of conjunctival lymphoma including classification, histology, staging, etiology, diagnostic methodology, recommendations for systemic evaluation, treatment, and prognosis. The purpose is to provide a comprehensive manual with an updated literature review of this entity for general and subspecialty ophthalmologists. In addition to this in-depth review, we report on the novel use of optical coherence tomography (OCT) technology in the diagnosis and monitoring of this disease.

## Review

A thorough PubMed search of articles published between January 1990 and July 2018 on the diagnosis and management of conjunctival lymphoma was performed. Searches included a combination of the following terms: “bendamustine,” “conjunctival lymphoma,” “ocular adnexal lymphoma,” “extranodal marginal zone lymphoma,” “optical coherence tomography,” “radiation therapy,” “chemotherapy,” “Chlamydia psittaci,” “doxycycline,” “interferon-alpha,” and “rituximab.” Pertinent articles were carefully reviewed and referenced in this paper.

### Classification

The histology and clinical stage of conjunctival lymphomas are the most important predictors of disease outcome.

#### Histopathology

The histological subtypes of lymphoma are divided into low- and high-grade categories **(**Table 1**)**. Conjunctival lymphomas are most commonly extranodal marginal zone lymphoma (EMZL) and follicular lymphoma (FL), both of which are generally low-grade. EMZL, previously known as mucosa-associated lymphoid tissue (MALT) lymphoma, constitutes approximately 80% of conjunctival B-cell non-Hodgkin lymphomas (NHL). EMZL is slightly more common in female patients and typically presents in the patient’s sixties [3, 5]. FL is the second most common conjunctival lymphoma subtype, comprising approximately 8% of tumors. It also commonly presents in the seventh decade of life [3, 6].

**Table 1:** Epidemiology of histologic subtypes of conjunctival lymphoma

Histologic subtype	Histologic grade	Percentage of conjunctival lymphoma	Gender predilection	Median age
EMZL	Low-grade	80%	Female	60s
FL	Low-grade	8%	Comparable	60s
DLBCL	High-grade	3%	Male	70s
MCL	High-grade	3%	Male	70s
T-cell NHL	High-grade	2%	Comparable	Insufficient data

*EMZL*= extra-nodal marginal zone lymphoma; *FL*= follicular lymphoma; *DLBCL*= diffuse large B-cell lymphoma; *MCL*= mantle cell lymphoma

Diffuse large B-cell lymphoma (DLBCL) and mantle cell lymphoma (MCL) are high-grade subtypes that each comprise approximately 3% of conjunctival lymphomas. These less common and faster-growing lymphomas most often occur in male patients in their seventies. Non-B-cell conjunctival lymphomas, derived from T lymphocyte or natural killer cells, are uncommon and carry a particularly poor prognosis, with a high predilection for systemic dissemination. Data on these lymphomas is scarce [3, 7–12].

#### Staging

Extent of regional nodal and distant systemic involvement at the time of diagnosis is part of the classification and staging of extranodal lymphoma. Most conjunctival lymphomas present as isolated disease without evidence of prior or concurrent systemic dissemination (67–90%) [3, 13–19]. The incidence of systemic disease is less frequent with low-grade subtypes than with high-grade subtypes [19–23]. Of the high-grade conjunctival lymphomas, approximately 25% of patients with DLBCL and 50% of patients with MCL have evidence of systemic lymphoma at the time of diagnosis. Up to 80% of T-cell NHL in the conjunctiva arise as secondary lymphomas from a distant source [3]. With any subtype of conjunctival lymphoma, long-term follow-up is key as systemic disease may develop months or years after the initial diagnosis [7, 15, 24].

### Etiology

The etiology of conjunctival lymphoma remains elusive. Predisposing factors that have been linked to the development of OAL include: immune deficiency or dysfunction, autoimmune conditions (e.g. Sjögrens, autoimmune thyroid disease, systemic lupus erythematosus, rheumatoid arthritis) [3, 5, 25–30], infectious etiologies, genetic mutations, and prior radiation exposure. Conjunctival lymphoma appears to develop as a result of chronic inflammation triggered by endogenous or exogenous antigens, leading to sustained proliferation that permits the potential for genetic mutations leading to eventual monoclonal B or T lymphocyte populations [3, 5, 25–27]. Chronic benign reactive lymphoid hyperplasia of the ocular adnexa may induce such a malignant transformation, but the incidence of lymphoma in these cases has been low [19, 25, 28, 29, 31].

Chronic antigenic stimulation associated with the pathogenesis of conjunctival lymphoma has also been associated with infectious agents including *Chlamydia psittaci*, *Chlamydia pneumoniae*, hepatitis C, and *Helicobacter pylori* [3, 26, 27, 30]. Some studies have named *C. psittaci* as a principal causative agent in EMZL of the ocular adnexa, while other studies have failed to show any evidence of association [17, 32–34]. The prevalence of *C. psittaci* appears to vary geographically [5, 26, 32, 35, 36]. Ferreri et al. reported hepatitis C virus seropositivity in 13% of patients with OAL [37]. While *H. pylori* has been explicitly linked to the development of MALT lymphoma in the stomach, similar affiliations between this infectious agent and lymphoma of the conjunctiva are suspected but not yet confirmed.

Several genetic translocations, inactivating mutations, and trisomies (in particular trisomy of chromosomes 3 and 18) have been linked with various subtypes of conjunctival lymphoma [3, 5, 18, 26, 30].

### Presentation

**Fig. 1:**
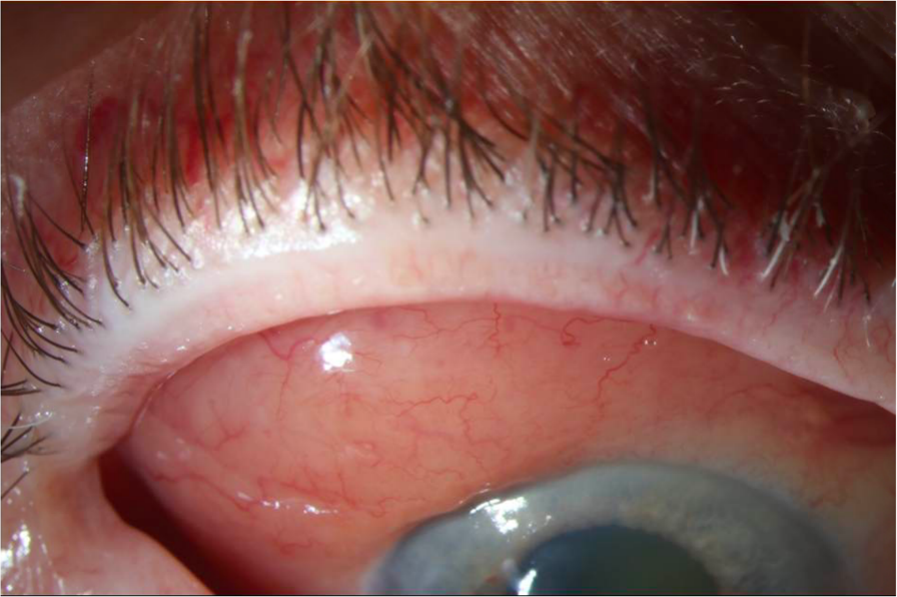
Slit lamp image of right eye with “salmon patch” conjunctival lesion in the superior temporal conjunctiva. The biopsy confirmed lymphoma

**Fig. 2:**
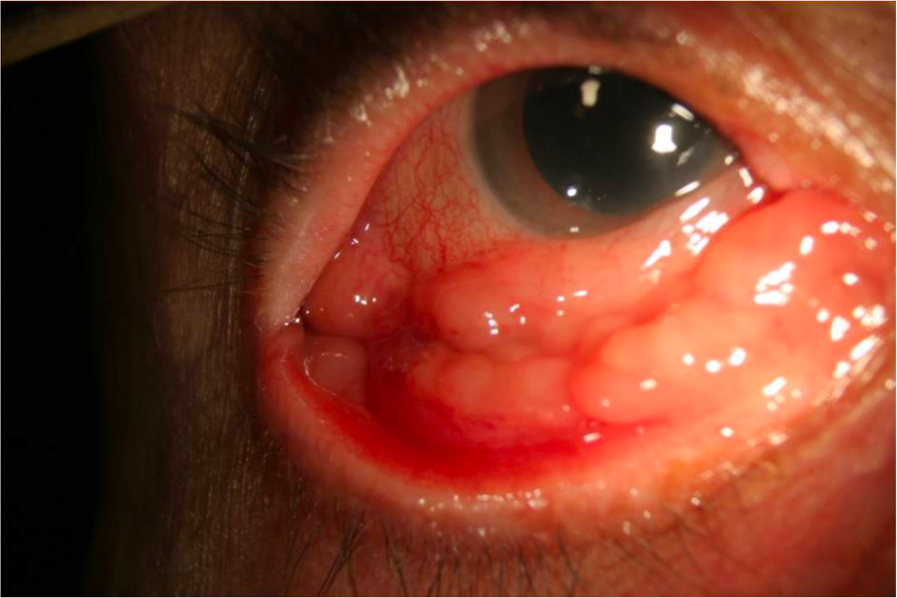
Conjunctival lymphoma in the right eye of a patient presenting as a chronic follicular conjunctivitis

Conjunctival lymphoma classically presents as a chronic, sessile, pink-colored sub-epithelial conjunctival mass described as a “salmon patch” **(**Fig. 1**)** [5, 27, 38]. Another presentation is that of a chronic follicular conjunctivitis **(**Fig. 2**)** [39]. Feeder vessels and rapid growth are not typically seen in lymphoma. Patients may be asymptomatic or note ocular irritation, redness, and, rarely, ptosis or exophthalmos (in cases with significant orbital involvement) [3, 16, 27, 40]. The relatively asymptomatic initial presentation, especially with low-grade subtypes, often leads to a delay in diagnosis [26]. B-cell NHL lesions are typically found in the conjunctival fornix or bulbar region, and less commonly in the caruncle or limbus [3, 40, 41]. In contrast, 30% of T-cell lymphomas occur in the limbus [3]. Bilateral lesions account for 10–15% of cases of conjunctival lymphoma overall [16, 42]. However, more than 50% of cases of the MCL subtype have bilateral involvement [3, 30].

### Diagnosis

The differential diagnosis includes: benign lymphoid hyperplasia, episcleritis, conjunctival amyloidosis, atypical pterygium, amelanotic melanoma, pyogenic granuloma, chronic conjunctivitis, and, rarely, squamous cell carcinoma or papilloma.

#### Optical coherence tomography

**Fig. 3:**
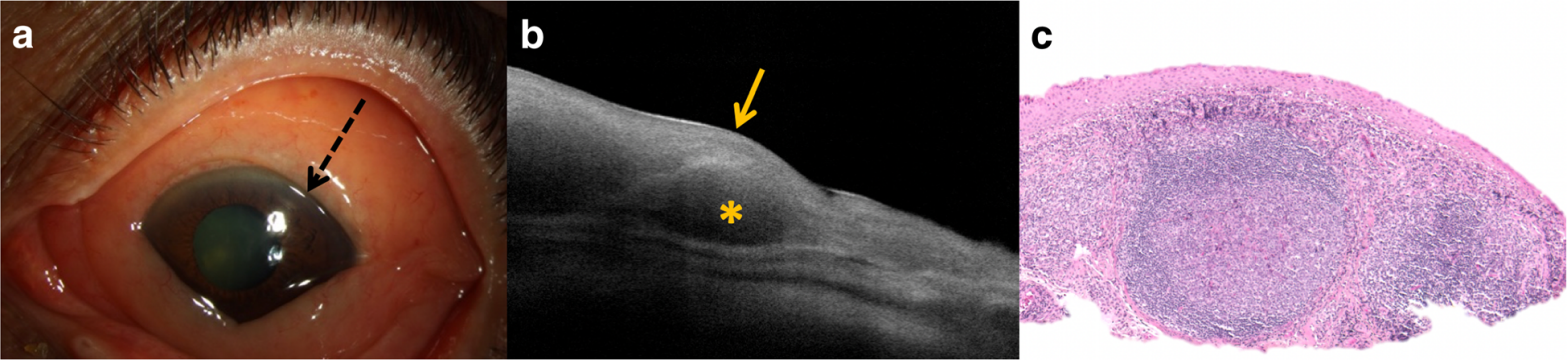
Clinical, high-resolution optical coherence tomography (HR-OCT), and pathological findings in conjunctival lymphoma. **a**. Slit lamp image of a diffuse conjunctival infiltrate in the bulbar conjunctiva of left eye **b**. HR-OCT revealing normal epithelium (arrow) and classic features of conjunctival lymphoma which include a hypo-reflective, homogenous subepithelial mass (*). **c**. Histopathologic examination discloses intact mucosal epithelium overlying lymphoid follicles present within the substantia propria with surrounding fibrous tissue corresponding to that present in the anterior segment HR-OCT. (Hematoxylin-eosin; original magnification 40 ×)

Recently, a novel approach has been introduced in the diagnosis of ocular surface tumors with the help of high resolution anterior segment optical coherence tomography (HR-OCT) [38, 43–45]. The use of HR-OCT in the evaluation of patients with conjunctival lesions is rapid and non-invasive, and the results can easily be interpreted and utilized [46]. OCT generates a cross-sectional image of tissue layers by compiling multiple interference patterns from light reflected on the ocular surface [38]. Characteristic findings of conjunctival lymphoma on OCT have been determined by several studies in the past decade. Using HR-OCT imaging, the lesion is identified as a hypo-reflective, homogenous subepithelial mass that appears to be composed of monomorphic, stippled, hypo-reflective dots **(**Fig. 3**)**. Epithelial appearance and thickness is normal in lymphoma cases. The lesion may be surrounded by a hyper-reflective band of substantia propria, which likely represents conjunctival tissue displaced by the underlying mass. While HR-OCT cannot distinguish benign reactive lymphoid hyperplasia from lymphoma, there are visible differences between lymphoma and other subconjunctival infiltrates. For example, the distinctive dark, monotonous ‘dots’ of conjunctival lymphoma are differentiated from the hyper-reflective ‘lines’ within the subconjunctival mass that characterize amyloid infiltrate [38, 45].

**Fig. 4:**
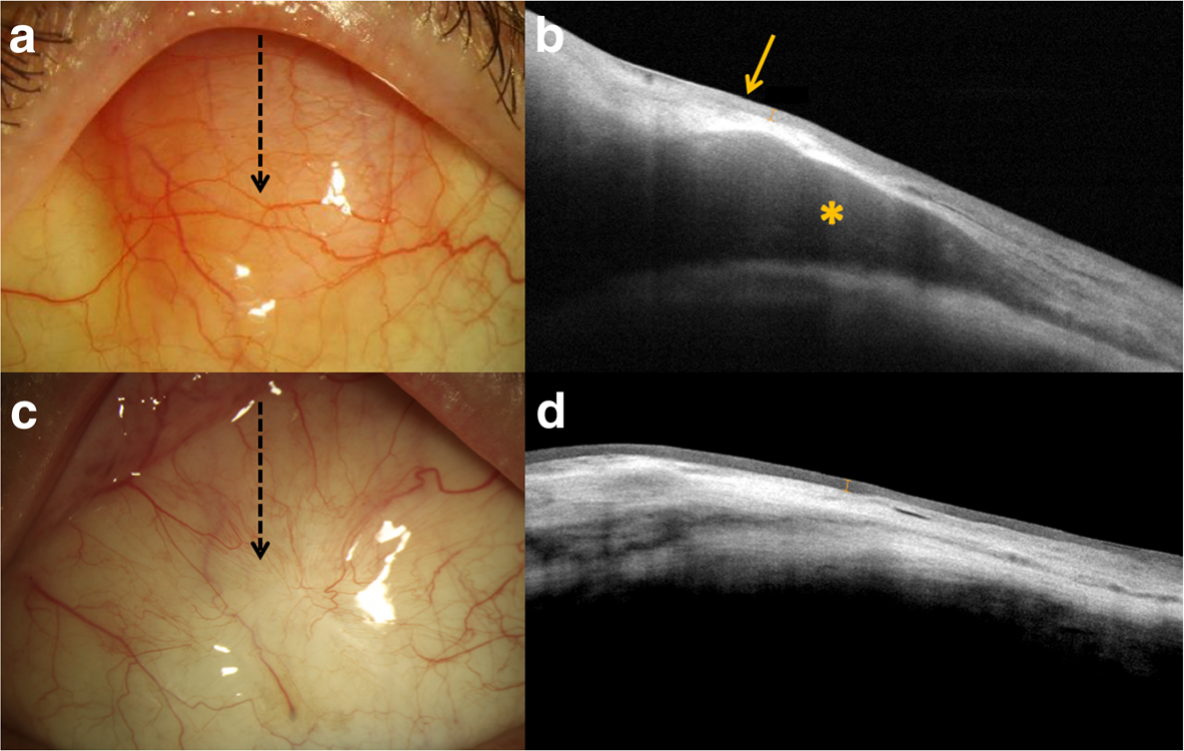
Clinical and high-resolution optical coherence tomography (HR-OCT) findings of a patient with conjunctival lymphoma before and after external beam radiation (EBRT) **a**. Slit lamp image of “salmon patch” in the superior bulbar conjunctiva (arrow location of OCT scan). **b**. HR-OCT revealing normal epithelium (arrow) and a hypo-reflective, homogenous subepithelial mass (*) consistent with conjunctival lymphoma. **c**. After confirmation with biopsy and treatment with 20 sessions of EBRT, the tumor resolved (arrow location of OCT scan). **d**. The HR-OCT post-treatment confirmed the resolution of the tumor

Limitations of HR-OCT in the diagnosis of ocular adnexal lesions include difficulty scanning lesions of substantial thickness due to shadowing and poor detection of stromal invasion. However, HR-OCT has been recognized as an exceptional tool in the monitoring of disease resolution during treatment **(**Fig. 4**)**. Case studies have shown that normalization of conjunctival architecture on OCT correlates well with completion of treatment and lesion resolution. In some cases, HR-OCT detected residual thickening or evidence of disease that was not visualized on clinical examination after initial treatment was concluded [38, 43, 45].

#### Biopsy

Surgical biopsy and histopathological examination are necessary to establish the diagnosis of conjunctival lymphoma. It is important to perform fresh tissue processing for both flow cytometry and gene rearrangement studies as clinical examination and radiographic studies alone are unable to distinguish malignant lymphoma from benign lymphoid hyperplasia [26, 30, 38, 47]. A portion of the biopsy should also be placed in formalin for histopathology (hematoxylin and eosin staining) and immunohistochemical staining.

#### Systemic work-up

Once conjunctival lymphoma is diagnosed, the extent of systemic disease should be established with a complete work-up by an oncologist. Work-up typically includes: complete blood count (CBC), serum chemistry studies (including lactate dehydrogenase (LDH)), computed tomography (CT) or magnetic resonance imaging (MRI) of the orbit, CT scan of other commonly affected areas (neck, chest, abdomen, pelvis), full-body positron emission tomography (PET) scan, and bone marrow aspiration and biopsy. The proper management of conjunctival lymphoma is determined by location, extent of periocular involvement, systemic staging of the disease, and general health of the patient.

#### Clinical staging

Clinical stage of conjunctival lymphoma is determined by Ann Arbor staging classification and the American Joint Committee on Cancer Tumor, Node, Metastasis (TNM)-based staging system for OAL **(**Table 2**)** [48–50]. The Ann Arbor staging system is determined by clinical presentation, imaging and laboratory tests, and initial biopsy reports. Each of the four stages is further categorized based on the presence of ‘B’ symptoms, defined as fever, night sweats, or weight loss of > 10% body weight over the last 6 months. The TNM system considers several factors that are not addressed by the Ann Arbor staging system. Primary tumor stage, T, is used to categorize the anatomic location of the tumor and tumor spread. Other specific factors designated by the TNM staging system are multiplicity and bilateralism of tumors, lymph node involvement, and distant spread at the time of presentation and diagnosis [49, 51, 52].

**Table 2:** Clinical staging of ocular adnexal lymphoma (OAL)

National Cancer Institute Working Formulation [48]	Low-grade	Small lymphocytic
		Follicular small cleaved cell
		Follicular mixed, small cleaved and large cell
	Intermediate-grade	Follicular large cell
		Diffuse small cleaved cell
		Diffuse mixed, small and large cell
		Diffuse large cell
	High-grade	Large cell, immunoblastic
		Lymphoblastic
		Small non-cleaved cell
Ann Arbor Staging [49]	Stage I	Involvement of a single lymph node region or extralymphatic site (IE)
	Stage II	Involvement of 2 or more lymph nodes, lymphatic structures, or extralymphatic regions alone on the same side of the diaphragm (IIE)
	Stage III	Involvement of lymph nodes on both sides of the diaphragm with localized extralymphatic (IIIE) or splenic (IIIS) involvement, or both (IIIES)
	Stage IV	Involvement of one or more organs or tissues outside the lymphatic system
		A: Without B symptoms B: Fever, night sweats, weight loss of > 10% body weight over the last 6 months
TNM Staging System [50]	T0	No evidence of lymphoma
	T1	Lymphoma involving the conjunctiva alone without orbital involvement
	T2	Lymphoma with orbital involvement ± any conjunctival involvement
	T3	Lymphoma with preseptal eyelid involvement ± orbital involvement ± any conjunctival involvement
	T4	Orbital adnexal lymphoma extending beyond orbit to adjacent structures, such as bone and brain
	N0	No evidence of lymph node involvement
	N1	Involvement of ipsilateral regional lymph nodes
	N2	Involvement of contralateral or bilateral regional lymph nodes
	N3	Involvement of peripheral lymph nodes not draining ocular adnexal region
	N4	Involvement of central lymph nodes
	M0	No evidence of involvement of other extranodal sites
	M1	Lymphomatous involvement in other organs recorded either at first diagnosis or subsequently

*TNM*= Tumor, Node, Metastasis

### Treatment

#### Treatment of isolated conjunctival lymphoma

**Fig. 5:**
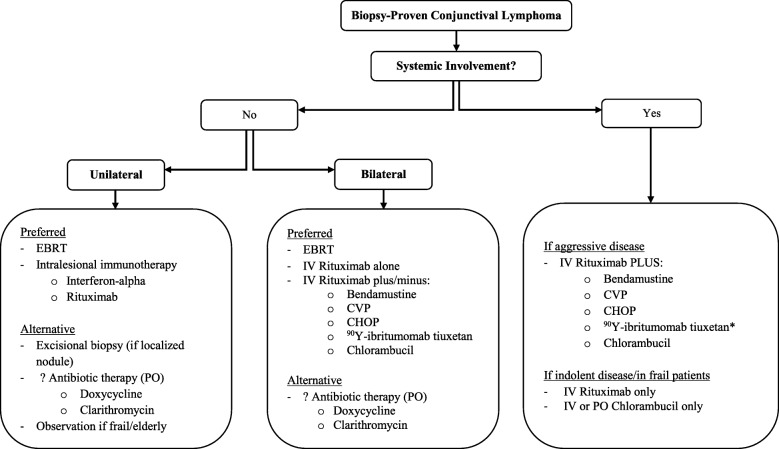
Approach to treatment of conjunctival lymphoma. EBRT: external beam radiation therapy; PO: per os (by mouth); IV: intravenous; CVP: cyclophosphamide, vincristine, prednisolone; CHOP: cyclophosphamide, doxorubicin, vincristine, prednisone. *If less than 25% bone marrow involvement

External beam radiation therapy (EBRT) is the gold standard treatment for lymphoma that is isolated to the conjunctiva or to the orbit including the conjunctiva, classified as Ann Arbor Stage I or T1N0M0 or T2N0M0 according to the AJCC criteria. Other less robustly studied but successful treatment options include local injection of immunotherapy and antibiotic therapy. In cases of bilateral OAL, occasionally systemic treatment is selected over bilateral external beam radiation. Surgical excision alone has shown higher rates of local and systemic recurrence as compared with the treatment options to be detailed below **(**Table 3**)** [53, 88]. Very rarely, cases of spontaneous regression of conjunctival disease after excisional biopsy have been reported [63, 89]. A watch-and-wait approach may rarely be chosen based on clinician and patient preference/age/health status in cases of unilateral conjunctival lymphoma of indolent histological subtype, but is not recommended due to the possibility of progression of ophthalmic disease as well as the appearance of systemic disease in the future [3, 61, 90–92] **(**Fig. 5**)**.

**Table 3:** Primary, isolated conjunctival lymphoma: Outcome, recurrence, and side effects of local treatment

Therapy type	Author	Year of publication	Number of eyes	Laterality	Histologic subtype or tumor grade	Response rate (CR + PR)	Follow up (months)	Local recurrence rate	Side effects
Radiotherapy	Baldini et al. [53]	1998	5	Unilateral	EMZL (100%)	100%	76 (median)	0%	Cataract (1 patient)
	Bhatia et al. [54]	2002	17	Unspecified	Unspecified	100%	Unspecified	Unspecified	Cataract, dry eye, corneal toxicity (unspecified)
	Bolek et al. [55]	1999	4	Unilateral	Low-grade (100%)	100%	12.6 (median)	0%	Ocular irritation, conjunctivitis, cataract (unspecified)
	Dunbar et al. [56]	1990	10	8 unilateral; 1 bilateral	Unspecified	100%	29.5 (median)	0%	Epilation of eyelashes, erythema of the eyelid, conjunctival injection; excessive tearing (25%)
	Erickson et al. [57]	1992	15	7 unilateral; 4 bilateral	EMZL (100%)	93%	Unspecified	Unspecified	
	Hasegawa et al. [58]	2003	9	Unilateral	EMZL (67%); DLBCL (11%); unspecified (22%)	100%	94 (median)	0%	Cataract (55.6%)
	Jereb et al. [31]	1984	5	Unilateral	DWDL (60%); NWDL (20%); DPDL (20%)	100%	22 (median)	0%	Slight erythema and conjunctivitis in 1 patient
	Kennerdell et al. [59]	1999	4	Unspecified	EMZL (100%)	100%		Unspecified	Mild xerophthalmia and chemosis (unspecified)
	Kuhnt et al. [24]	2003	1	Unilateral	EMZL	100%	144	0%	Cataract
	Lee, G-I et al. [60]	2018	121	37 unilateral; 42 bilateral	EMZL (100%)	100%	61.3 (median)	98%	Dry eye (26.6%); eye pain (5.1%); tearing (6.3%); cataract (6.3%)
	Lee, S-w et al. [23]	2002	4	Unspecified	EMZL (100%)	100%	31 (median)	0%	Conjunctivitis (100%)
	Liao et al. [61]	2002	12	Unspecified	Low-grade (83%), Intermediate-grade (17%)	100%	56.4 (mean)	0%	Lacrimal gland dysfunction (50%); cataract (25%)
	Martinet et al. [62]	2003	34	22 unilateral; 6 bilateral	Low-grade (100%)	100%	55 (median)	0%	Inflammatory reaction; cataract (unspecified)
	Matsuo et al. [63]	2004	6	4 unilateral; 2 bilateral	EMZL (100%)	100%	48 (median)	0%	Unspecified
	Pelloski et al. [64]	2001	11	9 unilateral; 2 bilateral	SLL (91%); SLP (9%)	100%	87.5 (median)	0%	Cataract (18%); diabetic retinopathy (9%); epiphora (9%)
	Shirota et al. [65]	2017	19	Unspecified	Unspecified	100%	32 (median)	0%	Cataract (unspecified)
	Smitt & Donaldson [13]	1993	20	10 unilateral; 5 bilateral	DSC (45%); DWDL (25%); FM (20%); F + DSC (5%); ALH (5%)	Unspecified	44 (median)	6.7%	Mild conjunctival irritation (unspecified)
	Stafford et al. [66]	2001	16	Unspecified	Unspecified	Unspecified	64.2 (median)	6.25%	
	Uno et al. [67]	2003	29	Unspecified	EMZL (100%)	100%	46 (median)	10%	Cataract, conjunctival irritation (unspecified)
	Vitu et al. [68]	1991	19	9 unilateral; 5 bilateral	Unspecified	100%	Unspecified	7% (bilateral recurrence in patient with bilateral disease)	Cataract (31.6%)
	Xicoy et al. [69]	2002	5	3 unilateral; 1 bilateral	EMZL (100%)	100%	50.5 (median)	0%	Conjunctivitis and epiphora (50%)
IFN-alpha	Blasi et al. [70]	2012	20	12 unilateral; 4 bilateral	EMZL (100%)	100%	65 (median)	15%	Temporary conjunctival chemosis and other ocular discomfort associated with injection; transient flu-like syndrome (100%)
	Cellini et al. [71]	1996	1	Unilateral	EMZL	100%	12	0%	
	Holds et al. [72]	2012	2	Bilateral	EMZL (100%)	100%	27	0%	Mild discomfort associated with injections, transient loss of appetite
	Lachapelle et al. [73]	2000	1	Unilateral	EMZL	100%	6	0%	Transient headaches and nausea; subconjunctival hemorrhage
	Lucas et al. [74]	2003	2	Bilateral	Likely EMZL	100%	18	0%	
	Ross et al. [75]	2004	2	Bilateral	Unspecified	100%	12	0%	Injection discomfort, mild flu-like illness
	Zayed et al. [76]	2013	1	Unilateral	EMZL	100%	10	0%	
	Zinzani et al. [77]	1997	4	Unilateral	EMZL (100%)	100%	32 (median)	0%	
	Zinzani et al. [78]	1999	4	Unspecified	EMZL (100%)	100%	47 (median)	0%	
Rituximab	Crespo et al. [79]	2014	2	Bilateral	EMZL	100%	9	0%	Dry eye
	Ferreri et al. [80]	2011	3	1 unilateral; 2 bilateral	EMZL (100%)	100%	11.5 (median)	0%	Ocular discomfort associated with injection (1 patient)
	Rodriguez Villa et al. [81]	2017	1	Unilateral	FL	100%	10	0%	
Antibiotic therapy	Abramson, Rollins, and Coleman [82] (PrevPak or Doxycycline)	2005	3	Unilateral	EMZL or unspecified low-grade (100%)	100%	21 (median)	0%	
	Danilko et al. [83] (Clarithromycin)	2013	1	Unilateral	EMZL	100%	0	0%	
	Ferreri et al. [84] (Doxycycline)	2006	14	Unspecified	EMZL (100%)	42.8%	Unspecified	21.4%	
	Govi et al. [85] (Clarithromycin)	2010	5	3 unilateral; 1 bilateral	EMZL (100%)	100%	27 (median)	0%	Unspecified
	Grünberger et al. [86] (Doxycycline)	2006	5	1 unilateral; 2 bilateral	EMZL (100%)	0%	9 (median)	N/A	
	Höh et al. [87] (Doxycycline)	2016	1	Unilateral	EMZL	100%	60	0%	

*CR*= complete response; *PR*= partial response; *EMZL*= extra-nodal marginal zone lymphoma [low-grade]; *FL*= follicular lymphoma [low-grade]; *DLBCL*= diffuse large B-cell lymphoma [high-grade]; *MCL*= mantle cell lymphoma [high-grade]; *DPDL*= diffuse poorly differentiated; *DWDL*= diffuse well differentiated; *NWDL*= nodular well differentiated; *DSC*= diffuse small cleaved; *FM*= follicular mixed; *ALH*= atypical lymphoid hyperplasia; *SLL*= small lymphocytic lymphoma; *SLP*= small lymphocytic lymphoma; plasmacytoid

##### External beam radiation therapy

EBRT has been considered the standard treatment for low-grade, isolated OALs for the past several decades. Five-year local control rates with radiotherapy alone in the treatment of OAL range from 89 to 100% [13, 54–57, 59, 60, 64, 66, 93–97]. The clinical target volume should include the entire conjunctival surface, from bulbar to the fornices to palpebral conjunctiva, while minimizing radiation to adjacent uninvolved areas of the eye and lacrimal gland. The entire orbit need not be included in the irradiated volume [5, 67, 98]. A dose range between 20 and 30 Gy is recommended as the primary treatment for indolent subtypes of isolated conjunctival lymphoma [30, 31, 56, 65, 98, 99]. Similarly, for indolent orbital lymphoma, doses of 24 to 25 Gy have been associated with satisfactory resolution of disease and long-term survival rates [5, 66, 93, 98, 100, 101].

The largest study to date on the use of radiation therapy for lymphoma isolated to the conjunctiva is a Korean study of 121 eyes (79 patients) with a median age at diagnosis of 38 years. Local control after 26 Gy radiation therapy was 98.1%. The 5-year survival rate was 100%; all but one of the relapsed cases were treated with re-radiation therapy. In this study, the radiation was delivered with five 2 Gy fractions per week [60].

Several studies have found associations between the site of disease, tumor grade, or histopathology of the lesion and long-term outcome of EBRT. In one retrospective study, relapse at distant sites after treatment completion was significantly higher in patients with lacrimal and soft tissue disease (51%) than in those with only conjunctival lesions (11%) [93]. Bolek et al. similarly found higher recurrence rates in cases with concomitant orbital adnexal lymphoma as compared to Stage I disease limited to the lid or conjunctiva [55]. Hasegawa et al. reported significantly longer 5- and 10-year overall and relapse-free survival rates of patients with indolent EMZL than in those with DLBCL [58].

Early minor complications of local radiation treatment include eyelid irritation and mild conjunctivitis. Long-term complications, which occur in up to 50% of patients, include dry eye syndrome (which can be severe), cataract formation, retinopathy, orbital fat tissue atrophy, and corneal ulceration [5, 93, 98, 102]. Lens shielding has been found to reduce the incidence of cataract formation in many studies [5, 13, 55, 56, 67, 88, 93, 98, 103]. Although the exact optimal dose of radiation in the treatment of OAL is subject to debate, doses above 35 Gy have had higher rates of post-treatment complications and morbidity in some studies [5, 93, 98]. In addition to a lower dose, smaller daily fractions may help to reduce radiation toxicity [49, 98].

Current literature reports possible therapeutic success with much lower doses of radiation than previously used. A recent retrospective review of 22 patients with EMZL, FL, or MCL of the ocular adnexa who underwent ultra-low-dose EBRT, 4 Gy delivered to the orbit(s) in two 2-Gy fractions on two consecutive days, revealed an overall response rate of 100%, after a median time of 3.73 months following treatment. Local control was 75% after 2 years [101]. Further studies with long-term follow-up are needed.

##### Immunotherapy

Interferon-alpha is a family of cytokines with anti-viral, anti-proliferative, and immunomodulatory functions. Interferons aid in cancer treatment by enhancing the immune response and directly affect tumor cells by increasing transcription of tumor suppressor gene p53, inducing apoptosis, and inhibiting angiogenesis [70]. Cellini et al. first documented the successful use of intralesional interferon-alpha in the treatment of conjunctival lymphoma [71]. Subsequently, its efficacy has largely been examined in case reports and small prospective studies. Interferon-alpha is typically administered as intralesional injections of 1 to 1.5 million international units (IU) in 0.25 mL, repeated three times weekly for a period of 4 to 6 weeks [70–73, 75–78]. Lucas et al. successfully treated a patient with bilateral lesions with 10 injections of 10 million IU given over a 4 week period [74]. Blasi et al. conducted the largest study to date on the outcome of intralesional interferon-alpha treatment of conjunctival lymphoma: 19 patients with primary EMZL of the conjunctiva and one patient with conjunctival lymphoma secondary to systemic disease that was in remission were treated with 12 doses of 1.5 million IU followed by another 12-dose course of 1 million IU of interferon-alpha. Local control was 85% after a median follow-up of 65 months [70].

Local side effects to interferon-alpha include temporary conjunctival chemosis at the site of injection and transient flu-like symptoms including fever, myalgia, and headache that may last for several hours after injection during the initial weeks of treatment [70, 72, 73, 75, 104]. In general, interferon-alpha has low toxicity and has not been associated with significant adverse effects in the localized treatment of conjunctival lymphoma.

Rituximab is a chimeric human-rodent monoclonal antibody that targets the surface antigen CD20, which is overexpressed on CD20-positive NHL B cells. Rituximab binding mediates complement-dependent cytotoxicity and antibody-dependent cellular cytotoxicity, and induces cellular apoptosis [102]. It is frequently administered intravenously in the treatment of a variety of systemic B-cell lymphomas, including cases of OAL with bilateral or systemic involvement [105, 106]. Intralesional rituximab therapy has been used in the treatment of primary B-cell cutaneous lymphoma with equivalent response rates, fewer adverse effects, and lower cost as compared to systemic rituximab treatment [107]. Recent case reports have commented on the efficacy of using intralesional injections in the treatment of relapsed and recurrent OALs [80, 81, 108, 109]. Intralesional rituximab is delivered as four weekly injections of approximately 1.5 mL followed by six monthly injections of the same, with the aid of topical anesthesia. Reports by Ferreri et al. and Crespo et al. describe recurrent cases of conjunctival EMZL, both unilateral and bilateral, that after undergoing several types of systemic treatment (e.g., IV rituximab, chemotherapy, antibiotic therapy, radiation) were successfully treated with intralesional rituximab without disease progression or recurrence at a range of 9 to 13 months [79, 80]. Demirci et al. describes a patient with a history of Sjögren syndrome with recurrence of bilateral lacrimal gland EMZL after completion of systemic rituximab therapy. The patient was then treated with intralesional rituximab and remained free from recurrence at 23 and 30 months of follow-up [110]. Rodríguez Villa et al. documented a case of previously untreated unilateral FL of the conjunctiva that resolved with intralesional rituximab [111].

No significant adverse ocular events have been reported secondary to administration of intralesional rituximab in the treatment of OAL. Some patients experience mild pain and local inflammation lasting less than 24 h after injection [80].

##### Antibiotic therapy

Despite the suspected link between OAL and *C. psittaci*, antibiotic therapy has been found to have variable efficacy and requires further investigation [32, 86]. Doxycycline is a well-tolerated therapeutic option that has been most successful when used in geographical regions that are known to have higher rates of *C. psittaci* infection. Typical dosing of doxycycline is 100 mg twice daily by mouth for 3 or 6 weeks.

In several Korean and Italian studies, doxycycline was shown to be a viable treatment option for T1N0M0 OAL, with two of the largest studies claiming 5-year progression-free survival (PFS) rates of 55 and 60.9% [36, 84, 112–114]. It has also been successful in smaller case studies in areas not typically associated with *C. psittaci* infection [82, 87]. Statistically significant improvements in response rate and survival have been associated with localized TNM stage, absence of absolute lymphocytosis, presence of absolute neutropenia, confirmation of *C. psittaci* infection, and treatment with a double course of doxycycline [84, 113, 114]. Of note, a large Korean retrospective study found conjunctival lymphomas to have superior response rates to doxycycline as compared to non-conjunctival lymphomas (OR = 11.8, 95% CI, 1.1–122.5; *P* = 0.038). In addition, the 2-year time to treatment failure (TTF) was 88% for conjunctival lymphoma, compared to 64% for non-conjunctival tumors [113].

The use of clarithromycin in the treatment of extranodal lymphoma has also been explored but reports are scarce [83, 85, 115]. Govi et al. published a study in which the patients with conjunctival disease had a superior response to a six-month course of clarithromycin; local control in these patients was 100% at a median follow-up time of 27 months [85].

#### Treatment of conjunctival lymphoma with systemic involvement

Systemic treatment is reserved for aggressive bilateral disease or conjunctival lymphoma accompanied by active systemic involvement. The recommended treatment for this is intravenous rituximab in combination with chemotherapy or other immunotherapeutic agents **(**Table 4**)**.

**Table 4:** Outcome, recurrence, and side effects of systemic treatment of conjunctival lymphoma

Therapy type	Author	Year of publication	Number of eyes	Laterality	Percentage of cases with preexisting or concurrent systemic disease	Histologic subtype	Response rate (CR + PR)	Follow up (months)	Local recurrence rate	Side effects
Chemotherapy	Baldini et al. [53] (Chlorambucil)	1998	1	Unilateral	0%	EMZL	100%	140	0%	
	Bellisi et al. [116] (Adriamycin, Bleomycin, Cyclophosphamide, Prednisone)	1982	5	3 unilateral; 1 bilateral	0%	DWDLL (50%); DPDLL (50%) (unspecified laterality)	100%	37 (median)	20%	
	Seker et al. [39] (CVP)	2010	2	Bilateral	0%	EMZL	100%	28	0%	
IV Rituximab	Annibali et al. [117]	2015	5	3 unilateral; 1 bilateral	0%	EMZL (100%)	100%	29 (median)	0%	VZV reactivation (1 patient)
	Celiker et al. [118]	2018	2	Bilateral	0%	EMZL	100%	22	0%	
	Ferreri et al. [119]	2005	3	2 unilateral; 1 bilateral	33%	EMZL (100%)	67%	5 (median)	67%	
	Nückel et al. [120]	2004	2	Unilateral	0%	EMZL (100%)	100%	31 (median)	0%	Reactivation of hepatitis B (1 patient)
	Rigacci et al. [121] (rituximab-Chlorambucil)	2007	4	Unilateral	0%	EMZL (75%); FL (25%)	100%	33 (median)	0%	
	Salepçi et al. [122]	2009	2	Bilateral	0%	EMZL	100%	16	0%	
	Sallak et al. [123] (Rituximab-Bendamustine)	2014	1	Unilateral	100%	EMZL	100%	36	0%	
	Tuncer et al. [124]	2015	6	Unilateral	0%	EMZL (83%); FL (17%)	100%	25	50%	
	Wall et al. [125]	2015	2	Bilateral	0%	FL	100%	15	0%	
	Zinzani et al. [126]	2005	1	Unilateral	0%	FL	100%	5	0%	
^90^Y-ibritumomab tiuxetan	Esmaeli et al. [127]	2009	5	Unilateral	0%	EMZL (100%)	100%	27 (median)	0%	Grades I-II pancytopenia (100%), mild fatigue, nausea, headache
	Oellers et al. [128]	2012	1	Unilateral	100%	EMZL	100%	3	0%	

*CR*= complete response; *PR*= partial response; *EMZL*= extra-nodal marginal zone lymphoma [low-grade]; *FL*= follicular lymphoma [low-grade]; *DLBCL*= diffuse large B-cell lymphoma [high-grade]; *MCL*= mantle cell lymphoma [high-grade]; *CVP*= cyclophosphamide, vincristine, prednisolone, *DWDLL*= diffuse well-differentiated lymphocytic lymphoma; *DPDLL*= diffuse poorly-differentiated lymphocytic lymphoma

Commonly used chemotherapeutic agents are chlorambucil and combined regimens such as CHOP [89, 92]. Due to the high risk of distant relapse associated with local radiation used in the treatment of intermediate and high-grade conjunctival lymphoma, adjuvant chemotherapy is recommended in complicated cases or aggressive histological subtypes (MCL, DLBCL, T-cell lymphoma) [13, 55, 89, 99, 129, 130].

##### Chemotherapy

Chemotherapy can be used as an adjunct to local treatment or as the sole therapy for OAL. It is the treatment of choice, typically in combination with rituximab, in cases of systemic disease, resistance to radiation, or contraindication of radiation therapy [89] **(**Fig. 5**)**. Data on the use of chemotherapy in patients with conjunctival lymphoma is limited. When used as a single agent or as part of combined therapy, it has produced varied results [53, 88, 103, 116, 131, 132].

Bendamustine is a chemotherapeutic drug with alkylating and antimetabolic properties. In 2008, it was approved for the treatment of both indolent and aggressive B-cell NHL after it was found to successfully treat NHL that had relapsed after primary treatment with rituximab or a rituximab-containing regimen in three independent phase II trials [133, 134]. Although further studies are needed to evaluate its treatment of OAL or conjunctival lymphoma specifically, there is robust evidence that bendamustine demonstrates excellent outcomes as both a single agent and in combination with rituximab [123, 133–136].

Chlorambucil, which is frequently used in combination chemotherapy regimens such as CVP (cyclophosphamide, vincristine, prednisolone) and CHOP (cyclophosphamide, doxorubicin, vincristine, prednisone), has a highly favorable toxicity profile. Complete response to chlorambucil has been observed in 67–100% of patients with OAL, but local relapse occurs in up to 29% of cases [137]. A study on OAL by Ben Simon et al. showed an overall response rate of 100% after an average of 4 courses of oral chlorambucil (average total dose 600 mg). Four patients (12%) had recurrence of disease; one was a case of local orbital recurrence while the other three developed extraorbital lymphoma disease. One of the relapsed patients died following a transformation to DLBCL [138]. A Korean study on EMZL of the ocular adnexa also reported an overall response rate of 100% to CVP. Seven cases (33%) showed disease recurrence at a median of 58 months after treatment, five local and two at extraorbital sites. The five cases of local failure obtained complete response after treatment with radiation therapy. Toxic effects associated with CVP in this study were neutropenia, anemia, elevated alanine aminotransferase, and peripheral neuropathy [139].

##### Immunotherapy

As discussed above, rituximab has cytotoxic effects on CD20-positive B cells and is the most commonly used immunotherapeutic agent for the treatment of lymphoma [102]. It is typically used in conjunction with other therapy in the treatment of conjunctival lymphoma with systemic involvement or with risk factors for systemic involvement. Typical treatment with single-agent rituximab consists of four to six consecutive weekly IV infusions at a dose of 375 mg/m^2^. It is very well tolerated. Most case reports in which rituximab was delivered in this manner in the treatment of newly diagnosed OAL revealed a 100% overall response rate [117–119, 122, 125, 126, 140]. However, a Ferreri et al. study calls into question the long-term efficacy of this treatment. In a 2005 study, four out of five recently diagnosed patients had local relapse at a median of 20 months after treatment. One of these patients also had systemic relapse with involvement of axillary lymph nodes and subcutaneous nodules [118]. Although follow-up was limited at a median of 29 months, Annibali et al. showed successful outcome maintenance in their study on six patients with EMZL-type OAL with an extended treatment course. Four patients (67%) obtained complete response and two patients (33%) obtained partial response. None of the patients had disease progression or recurrence [117]. Celiker et al. reported a case of bilateral conjunctival EMZL in which partial response was obtained after 6 cycles and complete response after 10. There was no recurrence after 22 months of follow-up [119]. These results contrast with those from a study by Tuncer et al., which revealed a complete response in only 4 of 11 reviewed cases of primary OAL. The remaining patients required additional radiotherapy due to partial response or recurrence of disease. In this study, though, of the six patients whose disease was isolated to the conjunctiva (5 EMZL and 1 FL), five achieved complete response and remained free of local disease for a median follow-up of 25 months [124].

Sullivan et al. demonstrated the usefulness of systemic rituximab treatment in OAL patients with preexisting or concurrent systemic disease. In this study, seven of eight patients responded to rituximab therapy. One of those had relapse of orbital disease at 26 months while the rest remained free of disease recurrence at a median of 17.5 months of follow-up. The eighth patient did not respond to rituximab treatment and passed away after progression of systemic disease [106]. Other case reports have also demonstrated long-term efficacy of systemic rituximab in the treatment of recurrent conjunctival disease [120, 122].

Rituximab is theorized to sensitize B cells to the effects of systemic treatment, and combination therapy with rituximab and conventional chemotherapy have been associated with higher response rates than chemotherapy alone in the treatment of NHL [102, 124]. Rigacci et al. used a combination of rituximab and chlorambucil in the treatment of nine newly diagnosed OAL patients, eight with EMZL and one with FL. Four of the patients had disease localized to the conjunctiva. Response rate was 100%; after a median follow-up of 25 months, no ocular toxicity nor disease progression was reported [121]. A larger, randomized study on patients with systemic MALT lymphoma (not of the ocular adnexa) showed patients who were treated with a combination of chlorambucil and rituximab had a significantly better median progression-free survival (*p* = 0.0119) than those patients who were treated with chlorambucil or rituximab alone [141].

##### Radioimmunotherapy

Radioimmunotherapy, in which monoclonal antibodies are used to deliver radioisotopes to the site of disease, has shown a better response than rituximab alone in some studies [142]. Yttrium 90-labeled ibritumomab tiuxetan (Zevalin®) is a radiolabeled anti-CD20 monoclonal antibody that is used in the treatment of refractory or relapsed low-grade B-cell NHL. It uses pure β emission to kill both target cells and nearby cells that may not express the antigen receptors via a bystander effect. This mechanism works independently of the host immune system. As is true with rituximab, ^90^Y-ibritumomab tiuxetan is well tolerated in patients. Transient pancytopenia often occurs in patients during the first 3 months following drug administration, sometimes necessitating transfusions. The estimated absorbed radiation to orbital soft tissues with ^90^Y-ibritumomab tiuxetan use is less than 3 Gy. Its use has not resulted in any of the ocular toxicities associated with external beam radiation treatment [102]. Other common side effects include fatigue, nausea, and headache [127]. Studies on ^90^Y-ibritumomab tiuxetan use in conjunctival and OAL are limited but have shown excellent response rates [127, 128, 142]. An established protocol by Esmaeli et al. dictates a treatment course that begins with intravenous administration of rituximab 250 mg/m^2^ prior to Indium total-body imaging. One week after this, patients are given a second dose of rituximab 250 mg/m^2^ IV, followed by ^90^Y-ibritumomab tiuxetan. Typically, patients with a platelet count of 100,000 – 149,000/mm^3^ are given 0.3 mCi/kg ^90^Y-ibritumomab tiuxetan, while patients with a platelet count above 150,000/mm^3^ are given a dose of 0.4 mCi/kg [127] **(**Fig. 5**)**.

### F. Prognosis

Ocular adnexal lymphoma has an overall 5-year survival rate ranging between 50 and 94%, depending on the grade of histologic subtype, TNM stage at diagnosis, and age of the patient [131]. Conjunctival lymphoma in particular has a good prognosis, with some studies demonstrating a 90% progression-or-recurrence-free population after 1 year of follow-up [3, 30].

The most important prognostic factor in conjunctival lymphoma is histological subtype of the lesion. Isolated cases of low-grade EMZL and FL are associated with the best outcome after treatment [3, 20, 49, 54, 58, 102, 143]. A 2016 retrospective study by Kierkegaard et al. on 263 patients with conjunctival lymphoma found the following 5-year survival rates: EMZL 97.0%, FL 82.0%, DLBCL 55.0%, and MCL 9.0%. In this study, most patients with localized disease were treated with EBRT with or without chemotherapy [144]. A 2018 study on EMZL of the ocular adnexa revealed patients with conjunctival disease to have a 66% 5-year progression-free survival and a 76% overall 5-year survival rate. Progression-free survival in this study was higher in conjunctival sites as compared to lacrimal gland and eyelid (50%), but lower than orbital sites (74%) [145].

Other clinical, laboratory, and various tumor biomarkers have been associated with prognostic effect. Established negative prognostic factors in the outcome of OAL include: age greater than 60–64 years [16, 62, 88, 146, 147], elevated serum LDH level [143, 147, 148], and increased blast percentage with positivity for tumor suppressor p53, and p21 and pRB positivity [26, 146]. Ferreri et al. reported that OAL patients with concomitant hepatitis C infection were more likely to have aggressive disease with lymph node and other extranodal organ involvement, higher relapse rates after treatment, and worse progression-free survival [37]. Coupland et al. investigated the prognostic value of cell-cycle associated markers in disease-free survival and lymphoma-related death. Tumor markers associated with higher risk for disseminated disease at some point during the observed clinical course included the lymphoma-associated transcription factor BCL-6, MUM1/IRF4 (multiple myeloma oncogene-1/interferon regulatory factor 4), and MIB1/Ki-67, a marker of cellular proliferation [146].

Advanced stage at the time of diagnosis also correlates with worse long-term prognosis of OAL [14, 20, 55, 62, 88, 99, 129, 143, 146, 147]. Lymphoma classified as Ann Arbor stage II-IV, indicating disease that has involvement beyond the extranodal site, is associated with earlier disease progression and/or relapse after initial treatment [16, 148]. One factor postulated to predict risk for disseminated disease is bilaterality [15, 67, 68, 102, 129, 149]. A 2001 Shields et al. analysis of 117 patients with lymphoid tumors of the conjunctiva found that 17% of patients with unilateral conjunctival involvement at the time of diagnosis had systemic lymphoma, while this number rose to 47% for those with bilateral conjunctival lesions [15]. Other studies have found no correlation between bilaterality of disease and worse prognosis [69].

Variation on outcome based on the site of OAL is debated. Many studies have found significantly better outcomes, including progression-free survival and overall survival, in patients with conjunctival lymphoma as compared to other ocular adnexal sites [62, 145, 148, 150]. However, other reviews have not found anatomic location to be an independent risk factor for disease-free survival or development of systemic disease [13, 49, 65, 67, 94, 146].

## Conclusion

Lymphoma is among the most common malignant conjunctival tumor. As the symptoms are often minimal, it is imperative for the general ophthalmologist to be alert for the characteristic “salmon patch” appearance of these neoplasms or to suspect lymphoma in individuals with unexplained chronic follicular conjunctivitis. Diagnosis is established via surgical biopsy with proper fresh tissue immunohistochemical studies. New imaging techniques with high resolution OCT can also help identify possible lymphoproliferative lesions as well as assist in the ongoing clinical evaluation during and after treatment. Systemic work-up and staging are critical to formulating the correct treatment plan. Both local and systemic treatments are available. The ophthalmologist should remain active in the management of possible ocular complications during lymphoma treatment. Long-term follow-up is necessary as systemic lymphoma may develop after many years.

## Data Availability

Not applicable.
